# The Future Challenge of Reactive Oxygen Species (ROS) in Hypertension: From Bench to Bed Side

**DOI:** 10.3390/ijms18091988

**Published:** 2017-09-15

**Authors:** Gabriele Togliatto, Giusy Lombardo, Maria Felice Brizzi

**Affiliations:** Department of Medical Sciences, University of Torino, 10126 Torino, Italy; gabriele.togliatto@unito.it (G.T.); giusy.lombardo@unito.it (G.L.)

**Keywords:** ROS, hypertension, mitochondria, redox signaling

## Abstract

Reactive oxygen species (ROS) act as signaling molecules that control physiological processes, including cell adaptation to stress. Redox signaling via ROS has quite recently become the focus of much attention in numerous pathological contexts, including neurodegenerative diseases, kidney and cardiovascular disease. Imbalance in ROS formation and degradation has also been implicated in essential hypertension. Essential hypertension is characterized by multiple genetic and environmental factors which do not completely explain its associated risk factors. Thereby, even if advances in therapy have led to a significant reduction in hypertension-associated complications, to interfere with the unbalance of redox signals might represent an additional therapeutic challenge. The decrease of nitric oxide (NO) levels, the antioxidant activity commonly found in preclinical models of hypertension and the ability of antioxidant approaches to reduce ROS levels have spurred clinicians to investigate the contribution of ROS in humans. Indeed, particular effort has recently been devoted to understanding how redox signaling may contribute to vascular pathobiology in human hypertension. However, although biomarkers of oxidative stress have been found to positively correlate with blood pressure in preclinical model of hypertension, human data are less convincing. We herein provide an overview of the most relevant mechanisms via which oxidative stress might contribute to the pathophysiology of essential hypertension. Moreover, alternative approaches, which are directed towards improving antioxidant machinery and/or interfering with ROS production, are also discussed.

## 1. Introduction

Hypertension, a common chronic condition, is a public health problem the world over [1,2]. Interestingly, more than 90% of cases do not have a known cause and current therapies simply aim to control the major symptom of hypertension: elevated arterial blood pressure. Despite extensive research, the intricate and multifactorial nature of the disease means that its pathophysiology is still unclear [3]. The pathophysiological mechanisms that contribute to high blood pressure are complex and include inflammation, remodeling, stiffness, calcification and associated atherosclerosis [4,5]. In this regard, the loss of appropriate endothelial functions, such as reduced vasodilation, increased vasoconstriction and loss of endothelial integrity, seem to play a major role. Oxidative stress, caused by excess ROS generation, decreased nitric oxide (NO) levels and reduced antioxidant capability in the cardiovascular, renal and central nervous systems, is a common feature. ROS regulate cellular processes, such as differentiation, proliferation, apoptosis, cell cycles and migration [6,7], under physiological conditions. ROS control endothelial function and vascular tone in the vascular system meaning that increased ROS production and/or weakened antioxidant defense mechanisms contribute to endothelium and smooth muscle cell (VSMC) dysfunction, which ultimately results in progressive organ failure [8,9,10,11]. The loss of redox homeostasis, characterized by diminished NO bioavailability and increased ROS production, also induces endothelial dysfunction, arterial remodeling and vascular inflammation [8,9,10,11]. ROS production can therefore lead to increased contractility, VSMC growth and apoptosis, monocyte migration, lipid peroxidation, inflammation and increased deposition of extracellular matrix proteins [7,11,12,13,14,15]. As originally reported by Redon and colleagues [16], increased levels of oxidative stress byproducts and decreased endogenous antioxidant enzyme action in peripheral mononuclear cells are common in patients with hypertension. Similar results have been obtained in differing experimental models of hypertension, including nephro-vascular hypertension [17,18,19] and Angiotensin II (Angio-II)-induced hypertension [20,21]. In addition, patients suffering from hypertension are connoted by a significantly higher level of plasma H_2_O_2_ than normotensive subjects [22,23]. Since these initial reports were published, intense efforts have been devoted to identifying the specific role that ROS play in these effects [24,25,26,27,28,29]. A “EU-ROS” position paper recently revised all these issues [30]. This concise review will discuss ROS action in vascular biology and its contribution to the development of endothelial dysfunction and vascular remodeling that leads to vascular tree fibrotic changes and hypertension.

## 2. Redox Signaling

Blood pressure homeostasis is regulated by a dynamic equilibrium of varying mechanisms [31]. In fact, a variety of molecules that belong to the “ROS group” and have different effects on cellular function are involved in the molecular events that control blood pressure [7,32,33]. The uncontrolled generation of ROS promotes oxidative stress and consequent DNA, protein and lipid damage, leading to cell injury and cytotoxicity [7,34]. Although ROS are generated during the reduction of molecular oxygen, the different chemical properties of individual ROS molecules have important implications and functions in cellular redox signaling. ROS usually include unstable free radicals, such as superoxide (O_2_^−^), and non-free radicals, such as hydrogen peroxide (H_2_O_2_), which are produced as intermediates in reduction–oxidation (redox) processes [7]. The life spans of O_2_^−^ and ^•^OH mean that these different ROS species can activate signaling pathways that sometimes have conflicting consequences. For example, it has been shown that, unlike H_2_O_2_, which has vasodilatory effects in a number of vascular diseases [35,36], O_2_^−^ acts as a vasoconstrictor and leads to endothelial dysfunction [8]. In addition, ROS byproducts are also derived from a number of metabolic processes, which include xanthine oxidoreductase activity, uncoupled NO synthase (NOS), nicotinamide adenine dinucleotide phosphate (NADPH) oxidases (Nox) and mitochondrial respiratory enzymes [31].

### 2.1. Xanthine Oxidase

Xanthine oxidase (XO) is a hepatic enzyme that catalyzes the production of uric acid, NO and ROS [37,38]. XO exists in two different forms: xanthine dehydrogenase (XDH) and XO [39]. Compelling evidence suggests that the cellular ratio of XO to XDH is critical to the development of atherosclerosis, endothelial dysfunction, nephro-vascular hypertension and cardiovascular disease in general [40]. Spontaneously hypertensive rats are characterized by the presence of elevated levels of endothelial XO and increased ROS production, which is associated with increases in XO activity and arteriolar tone [41]. Laakso et al. [42] have shown that xanthine oxidoreductase activity is associated with the development of hypertension-associated end-organ failure. Clinical data have demonstrated that the enzyme’s activity is increased both in hypertensive patients [40] and in patients with Angio-II-associated coronary disease [43]. Moreover, both endothelial XO [44] and plasma XO [45] activity are increased in human atherosclerotic plaques, suggesting XO-derived superoxide contributes to the development of hypertension-induced atherosclerosis [46,47]. Based on these data, circulating uric acid has been indicated as a potential oxidative stress biomarker [48,49].

### 2.2. Nitric Oxide Synthase and Arginase

Nitric oxide synthases (NOS) are a family of enzymes that catalyze the production of NO and citrulline from oxygen and l-arginine substrates [50]. In mammals, three isoforms of NOS (NOS1–3) encoded by distinct genes have been identified [50,51]. Two of them, NOS1 or neuronal NOS (nNOS), and NOS3 or endothelial NOS (eNOS) are constitutively expressed and synthetize NO in a calcium-dependent manner [52], while the latter enzyme, NOS2 or inducible NOS (iNOS), is a calcium-independent enzyme, which expression is regulated by inflammatory cytokines and bacterial toxins such as LPS [53] Unlike nNOS and eNOS, the expression of iNOS has been shown to induce detrimental effects by means of peroxynitrite-mediated apoptosis [54]. In fact, treatment with peroxynitrite scavenger prevented such damaging signals [54]. Moreover, data obtained in myocardial iNOS-overexpressing mice suggested a role of the enzyme in cardiac remodeling processes [55]. However, since these data have been questioned by a number of studies [56,57,58,59], the biological effects of iNOS are still controversial.

In the vascular system, NO production by eNOS is a fundamental mechanism of vascular homeostasis regulation [51]. Arginine is the foremost source for NO via NOS [60]. Indeed, four sets of enzymes are involved in arginine metabolism: arginase (ARG), NOS, arginine:glycine amidinotransferase (AGAT), and arginine decarboxylase (ADC). In particular, in the cytosol NOS metabolizes arginine to NO and l-citrulline; in the mitochondria ADC and AGAT metabolize Arginine into agmatine and creatine respectively; while both in the cytosol and in the mitochondria, ARG metabolizes arginine to urea and l-ornithine, the precursor of polyamines [60]. ARG consist of two distinct isoforms [61]. In particular, the isoform II of ARG is widely expressed [61]. In all expressing tissues, including the vascular system, ARG plays an important role in regulating the synthesis of proline and polyamines (putrescine, spermidine, and spermine) as well as NO [61,62,63]. This implies that, being arginine a limiting substrate for eNOS, ARG represents a crucial mediator of endothelial dysfunction. In this regard, an increased ARG activity has been linked to the development of high blood pressure in salt-loaded salt-sensitive Dahl rats [64]. Similar results were described in deoxycorticosterone acetate (DOCA)-salt hypertensive rats [65] and in endothelial cells of coronary arterioles isolated from renovascular hypertensive pigs [66]. Likewise, in spontaneously hypertensive rats, the increased expression and activity of aortic ARG has been associated with the attenuated relaxation responses to acetylcholine [67]. In fact, the non-selective arginase inhibitor, α-difluoromethylornithine, prevents the development of hypertension [67]. Finally, it has been shown that hemodynamic changes going along with the development of hypertension represent potent inducers of ARG mRNA expression in vascular cells [68].

All these data sustain the notion that reduced NO bioactivity, due to different mechanisms, is a common mechanism of endothelial dysfunction and the development of hypertension [69]. Indeed, the loss of NO bioavailability, due to a reduced synthesis or a defective anti-oxidant response contributes to endothelial dysfunction mainly because ROS production exceeds available antioxidant defense mechanisms. Abnormalities in its production and/or bioavailability go along with, and even precede, hypertension [69]. NO rapidly reacts with anion superoxide (O_2_^−^) to form peroxynitrite (ONOO^−^), which itself can cause vasoconstriction and lead to NOS uncoupling, lipid peroxidation and vascular damage [51,69,70]. Consistently, limited L-arginine availability promotes uncoupling of eNOS resulting in oxygen free radical formation [71].

Increased oxidative stress therefore contributes to the activation of the renin-angiotensin system, changes in glucose metabolism and renal impairment in hypertensive subjects. These effects lead to prolonged redox signaling and reduced NO bioavailability in renal microvasculature, resulting in increased afferent arteriolar tone and hypertension [72]. In this regard, there is some evidence to suggest that the mechanisms involved in reduced NO bioavailability can be attributed to decreased NO production and/or increased NO degradation [72]. Moreover, it has been reported that NO-mediated relaxation, in response to acetylcholine, is blunted in patients with hypertension or pre-hypertensive conditions [73]. The impairment of NO synthesis has also been reported in hypertensive patients with dysfunctional endothelia [74], diabetes mellitus [75], hypercholesterolemia [76] and cigarette smoke addiction [77].

Decreased NO production has been originally detected in pre-clinical models of hypertension [78,79] and diabetes [80,81]. In fact, marked renal and endothelial dysfunction, as well as myocardial infarction and dyslipidemia, have been described in knockout mice for n/i/eNOS isoforms [81].

Of particular interest, recent evidence sustains a role of NO in the maintenance of sodium balance and normotension [82,83]. This is consistent with the original observations that NO is synthesized by the macula densa (MD) and acts to suppress the tubuloglomerular feedback (TGF) [84,85,86]. An intriguing study by Wang et al. [87] demonstrated that a selective inhibition of NOS_1_β, a primary splice variant of nNOS expressed in the macula densa, in NOS_1_αKO and WT mice was associated with blunted glomerular filtration rate, diuretic and natriuretic responses after a high-salt (HS) diet [87]. Consistently, Lu and colleagues [88] demonstrated that deletion of NOS_1_β enhances TGF response and promotes the development of salt-sensitive hypertension. All together, these data suggest a specific role of NOS_1_β in renal hemodynamic auto-regulation and its potential dysregulation in salt sensitivity hypertension.

### 2.3. NADPH Oxidases

The group of mammalian NADPH oxidases is made up of seven members, Nox1 to Nox5 and Duox1 and -2, which are structurally similar; all contain FAD and NADPH binding sites, six transmembrane domains and bear two heme groups [89,90]. Depending on the amount of ROS produced in the vascular system, Nox proteins can have both beneficial and detrimental effects. While Nox proteins play an important role in the control of vascular tone and the formation of new blood vessels in physiological conditions [89,90], in hypertensive settings, it has been postulated that Nox expression and activity may lead to inflammation and fibrosis, as well as vascular remodeling [90,91,92]. Superoxide production via membrane NADH/NADPH oxidase activation in Angio-II-induced hypertension sustains the detrimental role of Nox in the pathogenesis of hypertension [21,93]. Rey and colleagues [94] have demonstrated that Nox inhibition attenuates blood pressure elevation in C57Bl/6Tac mice. In Angio-II–induced hypertension, a role for Nox proteins has also been reported in mice lacking p47phox. Indeed, it has been demonstrated that Nox proteins are a major source of O_2_^−^ and thus contribute to Angio-II-mediated hypertension [95]. In an intriguing work by Matsuno et al. [96], Nox1-null mice exhibited a reduction in ROS activity and a hypertensive response to Angio-II after seven days of treatment, demonstrating that the Nox1/NADPH oxidase-derived ROS in pressor response to Angio-II relies on reduced NO bioavailability. Accordingly, the smooth muscle-specific over-expression of Nox1 potentiates the hypertensive response to Angio-II [97], and similar effects were found in endothelial specific Nox2 overexpression [98]. Finally, ROS also regulate prostanoids resulting in vasoconstriction and reduced endothelium-dependent vasodilation [99,100]. Cyclooxygenase (COX), responsible for the formation of prostanoids, can produce ROS by itself via NADPH [99,100] and COX-derived prostanoids act as autocrine ROS inducers. This implies that ROS can act both upstream and downstream of the COX-prostanoid system [99,100,101] and strengthen the role that Nox proteins play in the development of hypertension through multiple mechanisms.

A fine investigation of NADPH oxidase-dependent ROS generation has been obtained by animal models of salt-sensitive hypertension. In knock-out mice lacking the gp91^PHOX^ gene, which encodes for a NADPH oxidase subunit, Ang II-induced hypertension was markedly attenuated [102]. Blunted arterial pressure was also found in salt-resistant SSp67phox null rats subjected to HS diet [103]. Moreover, NADPH oxidase upregulation and increase arterial pressure and urinary albumin excretion were found in MnSOD^−/−^, but not wildtype mice, subjected to HS diet [104]. These recent data support the original results in DOCA-salt hypertensive rats, where long term administration of the NADPH oxidase inhibitor, apocynin, significantly decreased systolic blood pressure and aortic O_2_^−^ production [105].

### 2.4. Mitochondria

Mitochondria mediate a variety of cellular processes, such as the maintaining of cellular redox status, cell survival and death, calcium homeostasis, while also participating in thermogenesis [106,107]. In physiological conditions, the electron transport chain (ETC) of mitochondria is in charge of efficiently carrying electrons from NADH through complexes I to V, the site of ATP generation. However, it has been demonstrated that hypertension may mediate mitochondria dysfunction [28,108]. Indeed, the mitochondria become dysfunctional under hypertension and mitochondrial superoxide radicals can have a significant impact on this disease. Furthermore, ROS from NADPH oxidase have been shown to enter the mitochondria under hypertension and promote electron loss and ROS production from the ETC [109]. A reduction in antioxidant enzymatic activity in patients with hypertension has also been reported [110]. There is therefore increasing interest in the role that antioxidants play in restoring mitochondrial function in this clinical setting [111]. Itani and colleagues [112] have recently shown that the inhibition of cyclophilin D (CypD), a regulatory subunit of the mitochondrial permeability transition pore (mPTP), which plays a critical role in mitochondrial ROS (mtROS) production, prevents Angio-II-induced hypertension in mice. Moreover, an interesting study by Widder [113] has shown that mitochondrial ROS contribute to Angio-II-induced myocardial hypertrophy, sustained vascular dysfunction, ROS generation and the development of hypertension. This would appear to suggest that mtROS contribute to the deleterious effects of Angio-II, which may be mediated via the direct interaction of Angio-II with mitochondrial components.

## 3. Oxidative Stress and Hypertension

While the rate of ROS production/clearance is balanced in physiological conditions, in oxidative stress conditions, the unbalance rate translates into increased ROS bioavailability and oxidative stress-mediated cellular damage [110,111,112,113,114]. Indeed, oxidative stress is a common feature of many vascular pathological conditions, such as atherosclerosis, hypercholesterolemia, hypertension, diabetes and heart failure. In the early 1990s, Nakazono et al. [115] demonstrated the existence of a relationship between ROS and hypertension in hypertensive rats by administering the anti-oxidant superoxide dismutase (SOD) mimetics. More recently, it has been shown that mice that lack ROS-producing enzymes display lower blood pressure than wild-type mice and that Angio-II infusion was unable to induce hypertension. Additionally, the ROS production-mediated expression of pro-inflammatory gene products [116] and the modulation of the early signal transduction pathways that are involved in the control of cell growth and death have been reported to contribute to hypertension-induced tissue damage [116,117]. Clinical studies have also demonstrated that patients with hypertension produced excessive amounts of ROS, exhibited higher levels of plasma H_2_O_2_ than normotensive subjects [16,118,119,120] and are characterized by unbalanced levels of antioxidant defense mechanisms [121]. This implies that oxidative signaling pathways are attractive targets with which to prevent the development of hypertension and possibly its complications [122,123]. However, the mechanisms involved in oxidative stress processes in hypertension are not yet fully understood. Decreases in NO bioavailability and the oxidation of biological active molecules, such as LDL, were the first well-characterized processes. Indeed, lipid abnormalities and oxidative stress also indirectly contribute to the development of vascular injury and the early phases of renal disease by stimulating mesangial cell (MC) proliferation [124]. In this regard, our laboratory demonstrated that adhesion molecules, such as β4 integrin, can exert stringent control on Rac-1/ROS-mediated MC cycle progression when exposed to ox-LDL [125]. These data point to the crucial role that Nox-activity-generated ROS play in the progression of kidney injuries that lead to hypertension. The oxidation of membrane fatty acids can lead to the formation of F2-isoprostanes, which are present in the blood of patients with diabetes and/or hypercholesterolemia. It is worth noting that plasma F2-isoprostanes are increased in animals with experimental hypertension and in humans with nephron-vascular hypertension [126,127]. Intriguing studies have recently demonstrated the presence of increased ROS generation in pulmonary arterial hypertension (PAH) patients [128,129], suggesting that ROS contribute to abnormal endothelial proliferation and PAH development. Moreover, it has been reported that Nox activity and expression are upregulated in animals and humans with PAH, suggesting that Nox-derived ROS may contribute to the development of PAH [130,131]. All the above data support the notion that the development of hypertension is associated with and, more importantly, could be caused by oxidative stress. This concept has been proven using a genetic approach, which reduces the generation of ROS, and using the overexpression of antioxidant enzymes [132]. The targeting of ROS and the increasing of NO bioavailability using specific compounds may well be a means of treating hypertension and a challenge for the future.

## 4. Antioxidant Therapies in Hypertension

Antioxidant defense systems in biological systems adapt themselves to the changing levels of oxidants in order to maintain an oxidant-antioxidant balance. Natural defenses against ROS include enzymatic and non-enzymatic systems. Non-enzymatic antioxidants include ascorbic acid (vitamin C) and α-tocopherol (vitamin E), and histidine dipeptides. Vitamin C and E were found to inhibit LDL oxidation, via ROS scavenging and by improving NO bioavailability [133,134]. Enzymatic antioxidant systems include enzymes such as superoxide dismutase, glutathione peroxidase and catalase. Compelling evidences have shown inverse correlations between the various circulating antioxidants and hypertension [135,136,137]. In fact, hypertensive subjects have reduced activity and/or decreased antioxidant enzyme content, including that of SOD, glutathione peroxidase and catalase [137,138,139,140]. Reducing oxidative damage by scavenging ROS with antioxidants should therefore ameliorate vascular injury and prevent the increase of blood pressure.

### 4.1. Clinical Studies

Antioxidants are molecules that can donate electrons and/or hydrogen atoms to oxidants, prevent the initiation of ROS chain reactions, remove free radical intermediates and inhibit other oxidative reactions [7]. The ability of antioxidants to trap ROS makes them capable of reducing oxidative damage and might possibly control blood pressure [141,142]. Vitamins C and E have been used as a therapeutic approach to decreasing oxidative stress and reducing blood pressure by acting on NADPH oxidase and NO bioavailability [143]. The oral administration of vitamin C has been shown to improve endothelium-dependent vasodilatory responsiveness by enhancing antioxidant status and reducing blood pressure in patients with hypertension [143,144]. XO inhibitors have also been used and allopurinol has, in fact, been investigated in clinical studies involving patients with hypertension [145]. Allopurinol administration was associated with improved cardiovascular outcomes when compared to matched non-exposed controls, suggesting that allopurinol has a positive effect on cardiovascular events [145]. Unfortunately, interventional clinical trials with antioxidants have not been as effective as expected [146,147,148,149,150].

Such disappointing outcomes can, however, be partially explained by insufficient dosage and the timing of the delivery of active compounds to the sites where they are needed. It has also been reported that high doses of antioxidants can increase oxidative stress [151].

### 4.2. Future Therapeutic Challenges

Progress in the search for the best Nox inhibitors has been made [152]. In fact, rather than scavenging ROS with non-specific antioxidant drugs, targeting compounds that inhibit specific NOX actions have been suggested as being more effective in modulating ROS production and could become future antihypertensive therapy development options (see Table 1). One of the first NADPH inhibitors used in preclinical studies was diphenyliodonium, which is very potent but lacks specificity [153]. A naturally occurring NADPH oxidase inhibitor, apocynin, was found to blunt the development of hypertension and to prevent endothelial dysfunction in hypertensive rats when orally administered [154]. However, similar to diphenyliodonium, apocynin is aspecific and lacks selectivity [155]. The second-generation of NOX inhibitors are more specific and selective [94,156,157,158]. Several NADPH potential oxidase inhibiting compounds are currently under investigation, however, none of the available Nox inhibitors is ready for clinical application and preclinical studies are not yet completed for many of them [159,160,161,162,163]. Further studies are needed to better understand the relationship between hypertension and the role of NADPH oxidase, because Nox inhibitors’ ability to prevent the formation of ROS means they present considerable advantages over antioxidants, which act only to moderate the effects of ROS that have been already produced.

Moreover, we must also keep in mind the fact that the role of oxidative stress in priming hypertension in humans is still debated, even if oxidative stress biomarkers positively correlate with blood pressure in essential hypertension [32,41,78,108,119] and circulating SOD was recently found to be a marker of cardiovascular alterations in hypertensive and diabetic patients [164]. Our laboratory has recently found that the naturally occurring unacylated form of ghrelin (UnAG) is a potent antioxidant compound, which acts on both progenitors and mature endothelial cells. It interferes with Rac1 activation in progenitor cells, even in humans [165], and induces SOD2 expression in preclinical models of acute ROS generation in vascular cells [166,167]. We have no data on its efficacy in hypertension; however, the potent anti-oxidant effects of UnAG might be tested in essential hypertension for its clinical benefits or as an oxidative stress proof of concept.

## 5. Conclusions

**Figure 1:**
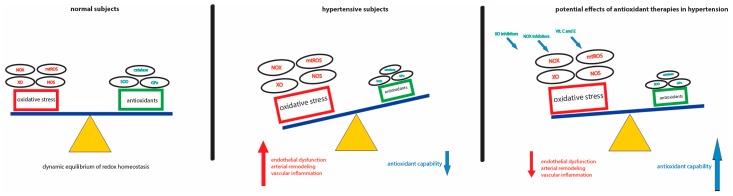
Schematic representation of ROS-mediated damage in hypertension and potential therapeutic approaches. The dynamic equilibrium of ROS production/clearance is balanced in physiological conditions (**left**). In hypertensive setting, the loss of redox homeostasis, characterized by a reduced antioxidant capability and increased ROS production, drives endothelial dysfunction, arterial remodeling and vascular inflammation (**middle**). Selective and specific ROS scavengers may be a novel approach to the treatment of hypertension-associated oxidative damage (**right**).

Compelling evidence from experimental and clinical studies indicates that oxidative stress might not only be the etiological cause of hypertension, but might also amplify the development of the disease. Despite the great enthusiasm for the efficacy of antioxidant compounds in preclinical studies, their translation into clinical benefits has failed due to possible confounding factors in patients with co-morbidity. Basic/clinical trials are therefore required to elucidate the role of antioxidants as novel anti-hypertensive therapeutics. One constant point that emerges from this review is that antioxidants would be more efficient in the early stages of the disease when deleterious ROS effects can be reversed. It is worth noting that recently identified inhibitors, which are directed against specific NOX isoenzymes, may be a novel approach to the treatment and prevention of hypertension-associated oxidative damage (Figure 1). However, the lack of evidence from large-scale randomized, controlled and multi-centric trials suggests that further studies are needed if we are to understand the specific contribution of NOX inhibition. To conclude, selective and specific ROS scavengers, rather than non-selective ROS inhibitors, should be developed to evaluate their long-term benefits in the clinic. However, it is always worth remembering that a suitable lifestyle should be recommended above all else since environmental factors are the main triggers of oxidative stress.

## Figures and Tables

**Table 1:** Data on novel XO and NOX inhibitors as therapeutics.

Biomarkers	In Vitro/Pre-Clinical/Clinical Sudies	Results Obtained	Reference
Allopurinol	clinical	Lower rates of stroke and cardiac events	[145]
diphenyliodonium (DPI)	in vitro	Abolished NADPH oxidase-mediated ROS formation, but also inhibited other flavo-enzymes such as NO synthase (NOS) and xanthine oxidase (XOD)	[153]
Apocynin	in vitro	Interfered with ROS detection but varied in efficacy and potency	[153]
Apocynin	pre-clinical	Completely abolished the development of spontaneous tone in endothelium-intact aortic rings (DOCA-salt hypertensive rats vs. SHAM-control rats)	[154]
Gp91 ds-tat	pre-clinical	Reduces Ang II–induced hypertension	[94,157]
ML171	in vitro	Blocks ROS-dependent formation	[157,158]
NoxA1ds	in vitro	Selective inhibitor of Nox1 activity and hypoxia-induced human pulmonary artery endothelial cell O_2_^−^ production	[161]
GKT137831	in vitro	Therapeutic potential in chronic hypertension-induced adverse cardiac remodeling	[162]
GKT137831	pre-clinical	Athero- and renoprotection in micro- and macrovascular complications	[163]

## Abbreviations

GTx	Glutathione peroxidase
mtROS	Mitochondrial ROS
NOS	Nitric oxide synthases
NOX	NADPH oxidase
SOD	Superoxide dismutase
Vit. C and E	Vitamin C and E
XO	Xanthine oxidase
